# Excretion of Cell-Free and Cell-Associated Zika Virus into Breast Milk of Infected Dams and Identification of Antiviral Factors

**DOI:** 10.3390/v14050851

**Published:** 2022-04-20

**Authors:** Sophie Desgraupes, Patricia Jeannin, Antoine Gessain, Pierre-Emmanuel Ceccaldi, Aurore Vidy

**Affiliations:** Institut Pasteur, Université Paris Cité, CNRS UMR 3569, Unité Épidémiologie et Physiopathologie des Virus Oncogènes, F-75015 Paris, France; sophie.desgraupes@pasteur.fr (S.D.); patricia.jeannin@pasteur.fr (P.J.); antoine.gessain@pasteur.fr (A.G.)

**Keywords:** mother-to-child transmission, breastfeeding, milk cells, antiviral, fatty acids

## Abstract

Zika virus (ZIKV) is a mosquito-borne RNA virus belonging to the *Flavivirus* genus of the *Flaviviridae* family. During the 60 years following its discovery in 1947, ZIKV caused little concern for public health as the associated infection was reported as mostly asymptomatic or inducing mild symptoms. However, since 2013, severe neurological symptoms have been associated with ZIKV infection, compelling the World Health Organization to declare a Public Health Emergency of International Concern. Among those symptoms, neurological birth defects may affect children born to mothers infected during pregnancy. Additionally, during the past 8 years, ZIKV transmission through breastfeeding has repeatedly been suggested in epidemiological studies and demonstrated on a mouse model by our team. To better understand the biological factors controlling ZIKV transmission through breastfeeding, we investigated the nature of the viral entities excreted in the breast milk of infected dams and evaluated viral transmission to breastfed pups. We show that both cell-free and cell-associated virus is excreted into breast milk and that ZIKV is efficiently transmitted to the breastfed pups. Additionally, we studied murine breast milk cell types, and identified a majority of mammary luminal cells. Finally, we investigated the effect on ZIKV infectivity of several breast milk components that are antiviral against different viruses such as lactoferrin (LF) and lactalbumin (LA), or free fatty acids (FFA). We showed no effect of LF and LA, whereas FFA inactivated the virus. These results bring new insight concerning the mechanisms of ZIKV transmission during breastfeeding and identify biological factors modulating it. These elements should be considered in risk assessment of ZIKV mother-to-child transmission.

## 1. Introduction

Zika virus (ZIKV) is an arthropod-borne virus (arbovirus) belonging to the *Flaviviridae* family and to the *Flavivirus* genus. It was first identified in 1947 in the Zika forest in Uganda and caused little concern for public health as ZIKV infection was reported as being mostly asymptomatic or associated with mild symptoms. During the following 60 years, only 14 human cases were reported in Southeast Asia and Africa [1]. In 2007, the first Zika fever outbreak ever described occurred in Yap islands, resulting in the infection of approximatively 5000 inhabitants (73% of the population) [2]. An even larger outbreak occurred in 2013–2014 in French Polynesia, causing approximatively 28,000 estimated cases of infection [3]. Two small outbreaks occurred that same year in New Caledonia where 1400 cases were described and in the Cook Islands where over 900 cases were reported [4]. Following the introduction of ZIKV in Brazil in 2015 [5], a large outbreak happened causing 440,000 to 1,300,000 cases [6], thus compelling the World Health Organization to declare a “Public Health Emergency of International Concern”.

As 80% of ZIKV infected individuals remain asymptomatic, the number of cases estimated during these epidemics is undervalued. After a small incubation period, 20% of infected people developed mild symptoms such as fever, skin rashes, and joint pain. However, since 2013, severe neurological symptoms have been associated with ZIKV infection [7]. In adults, Guillain–Barre syndrome (GBS) is the most common neurological complication, but other pathologies have been reported such as sensory polyneuropathy, sensory neuronopathy [7], and meningitis [8]. During the French Polynesian outbreak, the risk of developing a ZIKV-related GBS was estimated to be 0.24 per 1000 cases of infection [9]. Neurological birth defects, called the congenital Zika syndrome, have also been associated to ZIKV infection: children born to mothers infected during pregnancy can develop severe microcephalies, ocular abnormalities, or show decreased brain tissue [10].

ZIKV is mostly transmitted to humans by *Aedes aegypti* mosquitoes but interhuman transmission can also occur during sexual intercourse or from mother-to-child in utero [11]. During the past 8 years, ZIKV transmission through breastfeeding has repeatedly been suggested in epidemiological studies [12] and has been demonstrated on a mouse model by our team [13]. Epidemiological studies have reported the presence of viral RNA (vRNA) and/or infectious particles in the breast milk of infected mothers as well as viral persistence in breast milk after clearance from the blood stream [12]. In addition, a case of secondary microcephaly was recently reported in a breastfed infant who was negative for ZIKV at birth and showed a normal head circumference. The newborn was exclusively breastfed and ZIKV was isolated in the mother’s breast milk [14], raising concern about the transmission of ZIKV during breastfeeding.

Breast milk is mainly composed of water and contains maternal cells, carbohydrates (e.g., lactose), fat, proteins, and minerals [15]. In human breast milk, both breast-derived and blood-derived cells are found. Blood-derived immune cells represent a small minority of the milk cells (<2% in mature human milk), whereas the vast majority come from the mammary gland (luminal, myoepithelial, progenitor, and stem cells). Among those, luminal and myoepithelial cells represent 98% in human milk [16]. The different components of breast milk can be separated by centrifugation into a cell pellet, the lactoserum and the cream fraction. The lactoserum, also called whey, is the liquid fraction rich in proteins, lactose, and minerals, whereas the cream fraction contains the milk fat globules (MFG). The latter are composed of a triple-layered milk fat globule membrane (MFGM) surrounding a triglyceride core [17]. The composition of breast milk varies with different factors such as the species, nutrition, or stage of lactation [15]. Indeed, during the first hours after birth, the breast milk is rich in immunological components and called colostrum, whereas transitional and mature milk produced in the following weeks become richer in nutritional factors and poorer in leukocytes.

Although protective components are transmitted to the newborn during breastfeeding, deleterious factors such as drugs or pathogens can also be passed on. For humans, three viruses are recognized as efficiently transmitted during breastfeeding: human immunodeficiency virus type 1 (HIV-1), human T-cell leukemia virus type 1 (HTLV-1), and cytomegalovirus (CMV) [12]. These milk-borne viruses are excreted in human breast milk in a cell-free (HIV-1 [18], CMV [19]) or cell-associated (HIV-1 [18], CMV [19], HTLV-1 [20]) form and transmitted to the infant, resulting in a chronic infection.

As breast milk components and viral entities are simultaneously present in the milk, their interaction could lead to an enhanced or decreased viral transmission to the newborn during breastfeeding. Indeed, some breast milk components such as prostaglandins [21] have been shown to be proviral, whereas others such as lactoferrin (LF), monolaurin, or free fatty acids [12] are antiviral. Breast milk has been suggested to have an antiviral effect on some arboviruses [22], but it was further shown that the antiviral effect on ZIKV was caused by the storage of breast milk samples at 4 °C [23]. The authors showed that the storage of the samples leads to an increase in the free fatty acid content [24] and suggested a role of the latter in viral inactivation. Although milk stored at 4 °C exerted an antiviral effect, fresh breast milk from healthy donors was then tested and shown not to inactivate ZIKV [25], raising alarm concerning its transmission during breastfeeding.

Although cases of probable transmission of ZIKV from infected mothers to breastfed newborns have been reported [12], other case reports showed a lack of transmission by breastfeeding [26]. Therefore, biological or genetic factors controlling the transmission should be investigated (viral load, viral form, immunological status, stage of lactation, etc.).

In this paper, by using an established murine model for ZIKV infection [27], we show the infectious nature of breast milk isolated from ZIKV-infected dams and we identify the nature of viral entities in breast milk as being both cell-free and cell-associated. We also determine that the majority of milk cells in murine breast milk are mammary epithelial cells. Finally, we investigate the effect of several breast milk components on ZIKV infectivity (LF, lactalbumin (LA), and free fatty acids) in order to better understand the biological factors regulating the transmission.

## 2. Materials and Methods

### 2.1. Animal Model

A129 mice (129S2/SvPas-Ifnar1tm1Agt) were used to carry out the experiments, a well-established model to study ZIKV infection. They were housed and bred at the animal facilities of the Institut Pasteur which are accredited by the French Ministry of Agriculture for breeding and performing experiments on live rodents.

### 2.2. Ethics Statement

Experiments on animals were performed in compliance with French and European regulations on care and protection of laboratory animals (EC Directive 2010/63, French Law 2013-118, 6 February 2013). All experiments were approved by the Ethics Committee #89 and registered by the French “Ministère de l’Enseignement Supérieur, de la Recherche et de l’Innovation” under the reference “APAFIS#16119-2018071314475930 v1” (date of approval: 19 November 2018). Use of genetically modified mice (A129) was approved by the institutional instances and the French “Ministère de l’Enseignement supérieur, de la Recherche et de l’Innovation” under the reference no. 2194 (date of approval: 6 October 2017).

### 2.3. Virus Strains and Cell Lines

All experiments were carried out using two ZIKV strains belonging to the Asian lineage (Brazil/2016, GenBank: KU991811; H/PF13, GenBank: KX369547), amplified in Vero E6 cells as previously described [28]. ZIKV suspension was added to Vero E6 cell monolayers at 37 °C and 5% CO_2_ for 2 h in DMEM supplemented with 2% fetal bovine serum (FBS, Gibco, Thermo Fisher Scientific, Waltham, MA, USA), 100 U/mL penicillin, and 100 μg/mL streptomycin (Gibco, Thermo Fisher Scientific, Waltham, MA, USA). After adsorption, the inoculum was removed and replaced by DMEM containing 2% FBS and 10 mM Hepes (Gibco, Thermo Fisher Scientific, Waltham, MA, USA). After 2 days of culture, supernatants were centrifuged at 500× *g* for 10 min and aliquoted for storage at −80 °C until titration by plaque forming assay. These supernatants were used as inoculum for animal infections and in vitro experiments.

Vero E6 cells (CRL-1586, ATCC) and Caco-2 cells (Clone TC7, SCC209, Sigma-Aldrich, Saint-Louis, MO, USA) were grown in DMEM medium supplemented with l-glutamine (Gibco, Thermo Fisher Scientific, Waltham, MA, USA), 10% FBS, 100 U/mL penicillin, and 100 μg/mL streptomycin. They were maintained at 37 °C and 5% CO_2_ for culture.

### 2.4. Mouse Infection and Sample Collection

For in vivo experiments, 6- to 13-week-old A129 females and males were housed and bred for mating according to animal welfare recommendations. Lactating A129 mice were infected subcutaneously from 1 to 11 days post-partum with 10^6^ PFU of the Brazil/2016 strain of ZIKV. Blood samples were collected at 3 days post-infection in tubes containing 15 mM EDTA (Invitrogen, Thermo Fisher Scientific, Waltham, MA, USA) and subjected to plaque forming assays and RNA extractions to monitor the mothers’ infection. For milk sample collection at 4 days post-infection, the dams were isolated from their pups for 2 h 30 to 3 h to allow the accumulation of milk in the mammary glands. To stimulate the ejection of milk from the alveoli into the lactiferous ducts, the dams were injected intra-peritoneally with 250 µL of ocytocine (Ocytovem, Ceva Santé Animale, Libourne, France) and put in contact with their pups during a few minutes. The dams were watched closely to let the pups start suckling but prevent them from drinking all the breast milk. After stimulation, the dams were anesthetized by intra-peritoneal injection with 100 µL of an anesthetic mix containing 5 mg/kg of xylazine (Rompun, Bayer, Leverkusen, Germany) and 80 mg/kg of ketamine (Imalgene, Boehringer Ingelheim, Ingelheim am Rhein, Germany). Breast milk was collected with a Pasteur pipette by massaging the mammary glands. To evaluate the transmission, spleens were collected from euthanized pups and subjected to RNA extraction and RT-qPCR.

### 2.5. Milk Fraction Isolation

To separate the different fractions of breast milk, whole milk samples were centrifuged at 5000× *g* for 15 min. The cream fraction, the whey, and the cell pellet were then separated and washed twice in PBS at 5000× *g* for 15 min in order to increase their purity.

### 2.6. Viral RNA Extraction

vRNAs were extracted from plasma samples (obtained by blood centrifugation at 2000× *g* for 15 min), whole milk, cream fractions, whey fractions, and spleens, using the QIAamp viral RNA mini kit (Qiagen, Hilden, Germany). Spleens were stored at −80 °C before vRNA extractions. Spleens were thawed in PBS and dissociated using the Bullet Blender Storm shredder (Dutscher, Bernolsheim, France) in the presence of 3.2 mm steel beads (Next Advance, Troy, MO, USA). Spleen suspensions were centrifuged at 2000× *g* for 10 min and the supernatants were used for vRNA extractions.

### 2.7. RT-qPCR

Reverse transcription was performed using random hexamers and the Maxima H Minus Reverse-Transcriptase kit (Invitrogen). Quantitative PCR was performed using 5 µL of template cDNA, with 10 μL of MasterMix (iTaq Universal SYBR Green Supermix, Biorad, Hercules, CA, USA), 500 nM of each ZIKV NS5-specific primer (Forward: 5′–AAR TAC ACA TAC CAR AAC AAA GTG GT–3′; Reverse: 5′–TCC RCT CCC YCT YTG GTC TTG–3′), and using the following program: 10 min/95 °C, followed by 40 cycles of 15 s/95 °C, 20 s/60 °C, and 30 s/72 °C (Mastercycler Eppendorf Realplex, Hamburg, Germany). Quantification analysis was realized using a standard curve of a ZIKV-encoding plasmid.

### 2.8. Viral Titration by Plaque Forming Assays

Vero cell monolayers were exposed to 10-fold dilutions of plasma, whole milk, cream, and whey samples in DMEM-2% FBS during 2 h at 37 °C and 5% CO_2_. After viral adsorption, the inocula were removed and replaced with DMEM-2% FBS-1.6% carboxymethyl cellulose. The cells were cultured for 5 days at 37 °C and 5% CO_2_. After three washes in PBS, Vero monolayers were fixed in 4% paraformaldehyde (PFA) for 15 min at room temperature (RT) and colored with crystal violet (Sigma-Aldrich, Saint-Louis, MO, USA).

### 2.9. Co-Culture

Infectious center assays were performed by co-culturing milk cells with Vero E6 cells after 3-fold dilutions of 1 million milk cells in DMEM-2% FBS for 2 h at 37 °C and 5% CO_2_. DMEM-2% FBS-1.6% carboxymethyl cellulose was added and the co-cultures were kept for 6 days at 37 °C and 5% CO_2_.

After three washes in PBS, Vero monolayers were fixed in 4% PFA for 15 min at RT and colored with crystal violet.

### 2.10. Immune Staining and Flow Cytometry

After washing the cell pellets from breast milk samples twice in PBS, anti-mouse CD16/CD32 antibodies (Invitrogen) were added for 20 min at RT in order to block mouse antibody recognition. They were diluted in MACS buffer (FBS-2% EDTA-0.5% BSA) at a ratio of 2:50. After a wash in PBS at 400× *g* for 4 min, the extracellular antigens were stained for 1 h at RT in the dark (ratio 1:50 for antibody:MACS) as follows: anti-mouse CD45 stained with eF450 (Invitrogen), anti-mouse CD24 stained with APC (Invitrogen), and anti-mouse CD49f stained with PE (Invitrogen). The following steps were all performed in the dark. After two washes in PBS, cells were fixed using 4% PFA for 15 min at RT. After two washes in PBS, cells were resuspended in PBS-10% cell fix (BD Biosciences, Franklin Lakes, NJ, USA).

### 2.11. Cytotoxicity Assays

Vero or Caco-2 cells were incubated with DMEM-2% FBS, dimethyl sulfoxide (DMSO), or different concentrations of free fatty acids, LF or LA for 2 h at 37 °C. Cell layers were washed and cultured in DMEM-2% FBS at 37 °C and 5% CO_2_. Cell viability was assessed at 48 h of culture by an MTT colorimetric assay (thiazolyl blue tetrazolium bromide, Sigma-Aldrich). Cytotoxicity results can be found in Supplementary Figures S1 and S2.

### 2.12. Viral Particle Incubation with Lactoferrin, Lactalbumin, Ethanol, or DMSO

ZIKV particles were incubated with DMEM-2% FBS or different concentrations of LF, LA, ethanol, or DMSO for 1 h at 37 °C before infection at a multiplicity of infection (MOI) of 1. ZIKV infectivity was assessed using the Caco-2 cell line as, physiologically, ZIKV and breast milk are in contact with the newborn’s intestinal barrier. Viral inocula were added to Caco-2 monolayers in DMEM-2% FBS for 2 h at 37 °C and 5% CO_2_. The inocula were replaced by DMEM-2% FBS and the cells were cultured at 37 °C and 5% CO_2_ for 48 h. Cell layers were washed twice in PBS and subjected to vRNA extraction and RT-qPCR as described above.

### 2.13. Viral Particle Incubation with Free Fatty Acids

Viral inocula were incubated at 37 °C for 0 h, 1 h, 4 h, or 6 h and titrated in triplicates by plaque forming assays as described below (Table 1 and Table 2).

### 2.14. Cell Incubation with Free Fatty Acids, Lactoferrin, or Lactalbumin

Caco-2 cells were incubated with DMEM-2% FBS or different concentrations of free fatty acids, LF, or LA for 1 h at 37 °C before ZIKV infection (MOI 1). Viral inocula were added to Caco-2 monolayers in DMEM-2% FBS for 2 h at 37 °C and 5% CO_2_ then replaced by DMEM-2% FBS. The cells were cultured at 37 °C and 5% CO_2_ for 48 h. Cell layers were washed twice in PBS and subjected to vRNA extraction and RT-qPCR as described above. The results can be found in Supplementary Figure S4.

### 2.15. Statistical Analysis

All statistical analyses were performed using Prism 9.1.0 software. The number of experiments and the statistical tests used are detailed in the legends of the figures and presented as mean ± SD.

## 3. Results

### 3.1. ZIKV Is Efficiently Transmitted to Breastfed Pups

**Figure 1:**
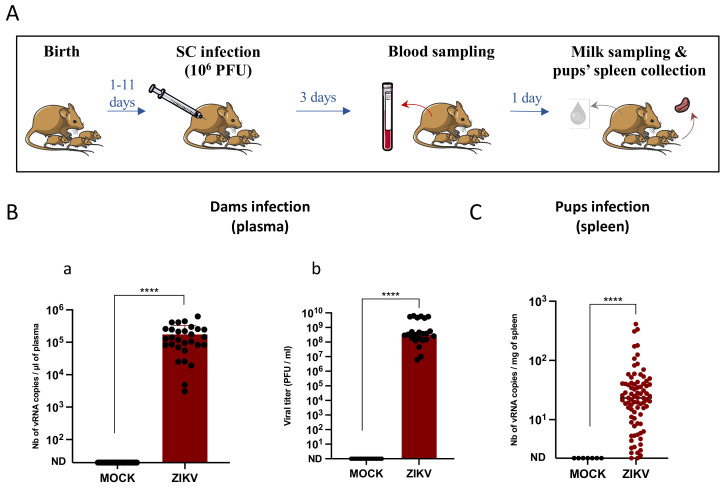
ZIKV is efficiently transmitted to breastfed pups. (**A**): Methodology of in vivo infections and sample collection for all mouse experiments. Female A129 dams were infected at 1 to 11 days post-partum with 10^6^ PFU of the Brazil/2016 strain of Zika virus (ZIKV) through the subcutaneous route (SC). Blood samples were collected from the dams at 3 days post-infection to assess ZIKV infection. Milk samples and pups’ spleens were collected at 4 days post-infection to investigate the presence of ZIKV. (**B**): Dam infection was assessed by RT-qPCR (**a**) (*n* = 33 mice) and plaque assays (**b**) (*n* = 23 mice) in the plasma. (**C**): Transmission was evaluated by RT-qPCR in the spleens of the pups (*n* = 83 pups). ****: Significantly different (*p* < 0.0001) by Mann–Whitney test (*p* < 0.05).

In order to study the transmission of ZIKV to breastfed newborn pups, 6- to 13-week-old female mice were mated with male mice. They were infected once with ZIKV (10^6^ PFU) through the subcutaneous route, between 1 and 11 days post-partum depending on the day of parturition. Infection was assessed by RT-qPCR and plaque assays performed on plasma samples collected at 3 days post-infection. Breast milk samples and pup spleens were collected at 4 days post-infection in order to investigate the presence of ZIKV (Figure 1A). Plasma samples were obtained by blood centrifugation and approximatively 10^5^ vRNA copies were detected per microliter of plasma and 10^8^ infectious particles per milliliter of plasma, confirming the infection of the dams with ZIKV (Figure 1B).

Pup infection was investigated by amplifying vRNA from pup spleens by RT-qPCR (Figure 1C). More than 90% of the pups that were breastfed by ZIKV-infected mothers were infected, with an average of 42.3 vRNA copies/mg of spleen.

### 3.2. Infectious Particles Are Excreted in Murine Breast Milk

**Figure 2:**
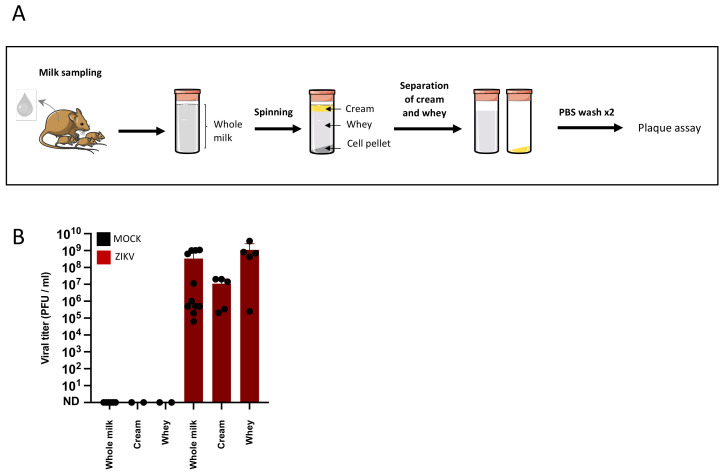
Infectious particles are excreted in murine breast milk. (**A**): Female dams were MOCK or ZIKV-infected with the Brazil/2016 strain as described in Figure 1. Breast milk samples were collected at 4 days post-infection and centrifuged to separate the cream fraction, the whey, and the cell pellet. The cream and whey fractions were isolated and further centrifuged twice with PBS to increase their purity. (**B**): The concentration of infectious viral particles in whole breast milk or breast milk fractions was determined by plaque assays (whole milk: *n* = 11; cream and whey: *n* = 5).

To evaluate the risk of transmission of ZIKV to the newborn, the presence of ZIKV was investigated in breast milk samples of infected dams. The dam infections and sample collection were performed as previously described and murine breast milk was harvested at 4 days post-infection (Figure 1A). To assess the infectious nature of the breast milk, the presence of infectious particles was investigated by plaque assays. The tests were performed either in whole breast milk samples or in fractions isolated by three spin-wash cycles (Figure 2A). To assess infectivity of breast milk, the latter was subjected to plaque assays and revealed 3.41 × 10^8^ PFU/mL (±standard deviation (SD) = 4.8 × 10^8^, 11 samples) of whole milk. The cream and whey fractions contained, respectively, 1.09 × 10^7^ (±SD = 10^7^, 5 samples) and 1.12 × 10^9^ (±SD = 1.4 × 10^9^, 5 samples) PFU/mL, demonstrating a high concentration of free infectious viral particles in murine breast milk (Figure 2B).

### 3.3. Murine Breast Milk Cells Are in Majority Mammary Epithelial Cells

**Figure 3:**
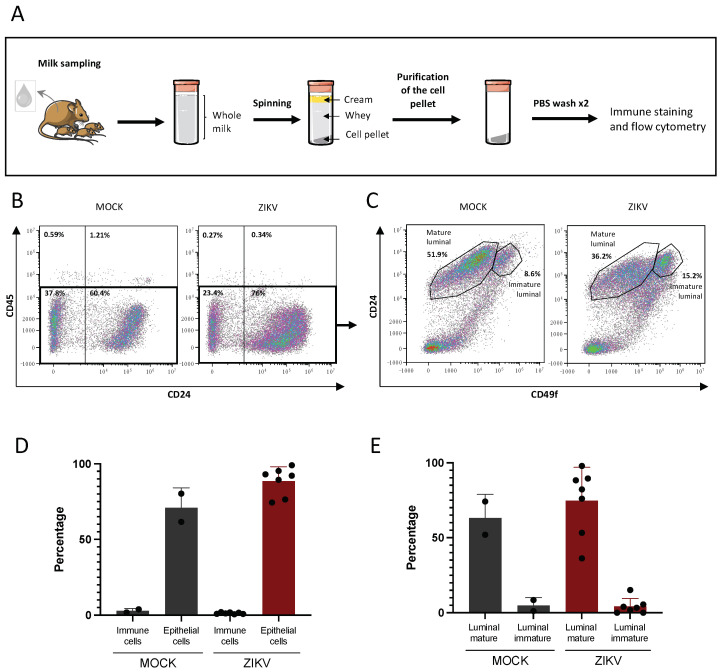
Breast milk cells of infected or non-infected mice are in majority mammary epithelial cells. (**A**): Breast milk samples collected from MOCK or ZIKV-infected dams with the Brazil/2016 strain at 4 days post-infection were centrifuged to separate the cream fraction, the whey, and the cell pellet. The cells were then subjected to two spin-wash cycles in PBS in order to eliminate any free viral particles left from the liquid fractions. Antigen expression was analyzed by immune staining and flow cytometry. (**B**): Anti-CD45 and anti-CD24 antibodies were used to detect and quantify immune and epithelial cells respectively. (**C**): CD49f expression was used to identify mammary epithelial cells among the CD45-negative cells determined in (**B**). The results of one experiment are presented (MOCK: *n* = 2, ZIKV: *n* = 7). (**D**): histogram representation of CD45-positive (immune) or CD24-positive (epithelial) cells in breast milk of MOCK or ZIKV-infected dams (MOCK: *n* = 2, ZIKV: *n* = 7). (**E**): histogram representation of mature and immature luminal mammary cells in breast milk, determined among the CD45-negative cells (MOCK: *n* = 2, ZIKV: *n* = 7).

To determine which cell types are present in murine breast milk, we isolated the cell content from murine breast milk samples collected as previously described (Figure 1A). After separation of the different breast milk fractions by centrifugation, the cell pellet was washed twice in PBS in order to eliminate free viral particles. Immune staining and flow cytometry were performed to determine the cell types present in murine breast milk (Figure 3A). To detect epithelial and immune cells, anti-CD24 and anti-CD45 antibodies were used, respectively. An average of 70.9% (*n* = 2) and 88.5% (*n* = 7) of epithelial cells were detected in the breast milk of MOCK-infected and ZIKV-infected dams, respectively. An average of 2.85% and 1.28% of immune cells were detected in MOCK (*n* = 2) and ZIKV (*n* = 7) breast milk samples (Figure 3B,D). To further characterize the epithelial cells detected in murine breast milk, CD49f expression was studied as it is differentially expressed by mammary epithelial cells. Indeed, luminal cells are CD24^high^/CD49f^+^ while myoepithelial cells are CD24^low^/CD49f^+^, and progenitor cells (immature luminal cells) are CD24^high^/CD49f^high^. In all breast milk samples, the majority of cells were CD24^high^/CD49f^+^ with an average of 68% in MOCK samples (*n* = 2) and 79.1% in ZIKV samples (*n* = 7). No clear population of CD24^low^/CD49f^+^ cells was detected in any of the samples, while some (MOCK: 2/2; ZIKV: 5/7) contained CD24^high^/CD49f^high^ cells ranging from 1.24% to 8.6% in MOCK samples and 1.88% to 15.2% in ZIKV samples (Figure 3C,E).

### 3.4. Infectious Cell-Associated ZIKV Is Excreted in Murine Breast Milk

**Figure 4:**
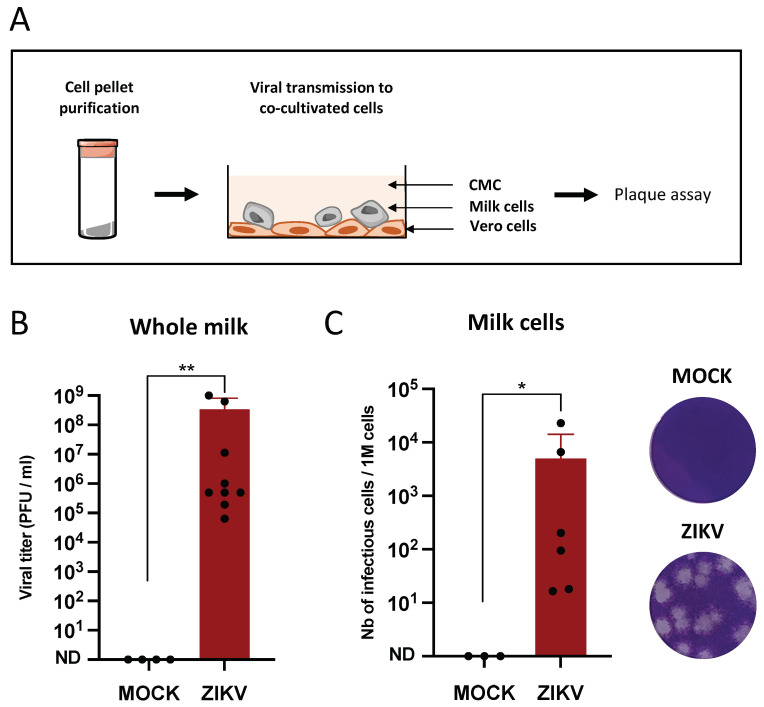
Infectious cell-associated ZIKV is excreted in murine breast milk. (**A**): Female dams were MOCK or ZIKV-infected with the Brazil/2016 strain as described in Figure 1. Breast milk samples were collected at 4 days post-infection and cell pellets were purified by three spin-wash cycles in PBS to eliminate free viral particles. (**B**): The viral titers in the same breast milk samples were determined by plaque assay on Vero cells. (**C**): For each sample (*n* = 6), duplicates of one million cells were serially diluted and co-cultured with Vero cells for 2 h at 37 °C. The cells were then co-cultured in medium containing carboxymethylcellulose for 6 days at 37 °C. After three PBS washes and PFA 4% fixation, the cell layers were stained with crystal violet and plaques were counted manually. * and **: Significantly different (**B**: *p* = 0.0015; **C**: *p* = 0.023) by Mann–Whitney test (*p* < 0.05).

In order to determine whether cells present in the milk are capable of transmitting the infection, co-cultures were performed between milk cells (purified as previously) and permissive target cells (Vero) in presence of carboxymethylcellulose (CMC). Vero cell infection was assessed by the observation of plaque formation after crystal violet staining (Figure 4A). In the same murine breast milk samples, the viral titers in whole milk (Figure 4B) and the number of infectious cells per million of total cells (Figure 4C) were determined. While viral titers in whole breast milk ranged from 6.33 × 10^4^ to 1.08 × 10^9^ PFU/mL (mean: 3.41 × 10^8^ PFU/mL) in samples collected from ZIKV-infected dams, the number of cells capable of transmitting the infection ranged from 1.65 × 10^1^ to 2.3 × 10^4^ cells/10^6^ cells (mean: 5 × 10^3^ cells/10^6^ cells), with a correlation between whole milk titers and the number of infectious cells (Spearman test, rho = 0.9).

### 3.5. Lactoferrin and Lactalbumin Are Not Antiviral against ZIKV

**Figure 5:**
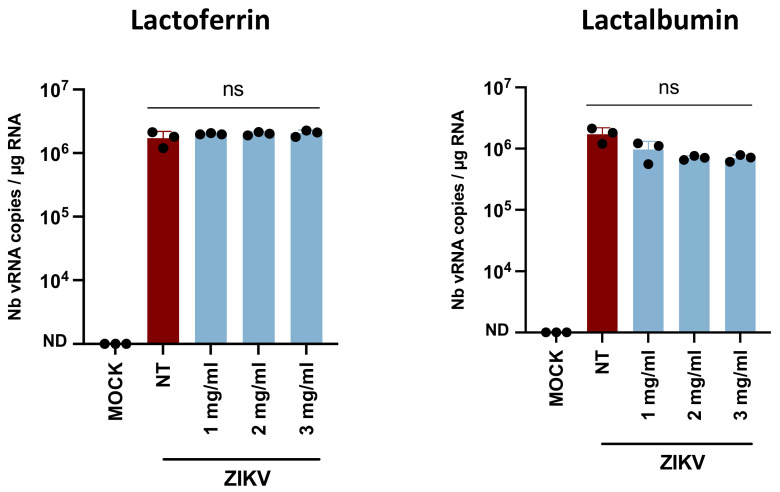
Lactoferrin and lactalbumin are not antiviral against ZIKV. ZIKV H/PF13 particles were incubated with culture medium (NT), lactoferrin, or lactalbumin (at physiological concentrations of human milk) for 1 h at 37 °C before infection of Caco-2 cells (MOI 1). At 48 h post-infection, cell layers were washed twice in PBS. After intracellular RNA extraction, viral RNAs were amplified by RT-qPCR (*n* = 3). ns: Non significantly different by Kruskal–Wallis test (*p* < 0.05).

In order to identify proviral or antiviral breast milk factors, the effect of LF and LA on ZIKV infectivity was studied by pre-incubation of different physiological concentrations (as reported in human breast milk [30,31]) of these factors with viral particles and subsequent infection of Caco-2 cells. The Caco-2 cell line was chosen to assess ZIKV infectivity because, physiologically, ZIKV and breast milk are in contact with the newborn’s intestinal barrier. The infection of the target cells was quantified by RNA extraction and vRNA amplification by RT-qPCR (Figure 5). Approximatively 10^6^ vRNA copies/µg of total RNA were detected in the Caco-2 cells with no significant difference between the non-treated, LF-treated, and LA-treated virus, suggesting the absence of effect of these factors on ZIKV infectivity.

### 3.6. Free Fatty Acids Are Antiviral against ZIKV

**Figure 6:**
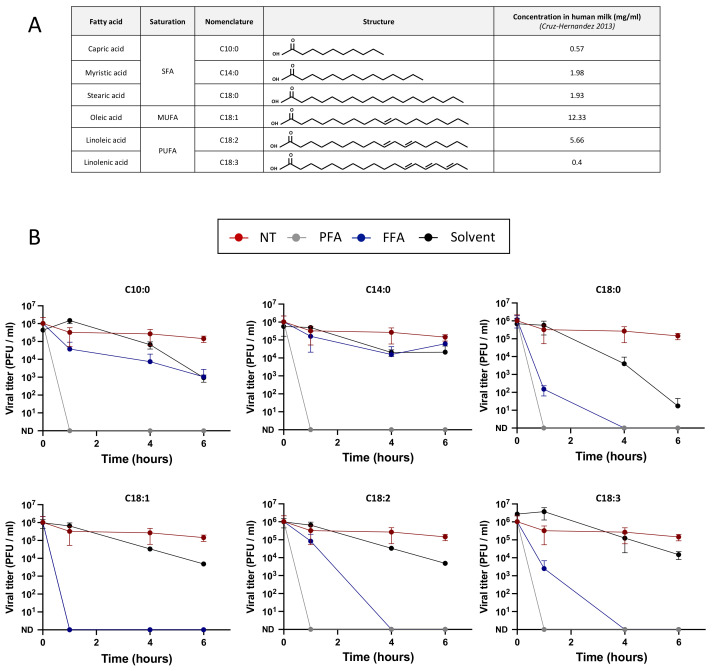
Free fatty acids are antiviral against ZIKV. (**A**): The fatty acids tested are either saturated fatty acids (SFA), mono-unsaturated fatty acids (MUFA), or polyunsaturated fatty acids (PUFA). Their nomenclature, structure, and abundance in human milk at 4 weeks after delivery (Cruz-Hernandez, 2013) are listed in the table. (**B**): To determine whether free fatty acids exert antiviral activity, 10^6^ PFU of ZIKV particles (Brazil/2016) were incubated with culture medium (NT), paraformaldehyde 4% (PFA), free fatty acids (FFA), or the solvent used to dilute them (solvent) for 6 h. At several time points (0 h, 1 h, 4 h, and 6 h), the viral titers were determined by plaque assays (*n* = 3).

In order to identify whether free fatty acids exert antiviral activity against ZIKV, as they have been shown to inactivate other enveloped viruses, viral particles were pre-incubated with six free fatty acids present in human breast milk (C10:0, C14:0, C18:0, C18:1, C18:2, and C18:3) before Vero cell infection and titration by plaque assay (Figure 6A). Viral particles were either pre-incubated with culture medium, PFA 4%, free fatty acids, or the solvent used to resuspend them (either ethanol or DMSO) (Table 1). The cytotoxicity assays of free fatty acids on target cells can be found in Supplementary Figure S1 and only non-cytotoxic concentrations were used for the antiviral assays (Table 2). No inactivation of the non-treated virus was observed over time, while pre-incubation with PFA 4% resulted in the inactivation of the virus in less than 1 h (first time point tested) as expected. The saturated fatty acids C10:0 and C14:0 exerted no effect on ZIKV infectivity for up to 6 h of incubation while C18:0 became antiviral after 4 h (Figure 6B). The mono-unsaturated fatty acid C18:1 inactivated ZIKV in less than 1 h of incubation, while the poly-unsaturated fatty acids C18:2 and C18:3 inactivated ZIKV in 4 h (Figure 6B), suggesting a role of the unsaturations in viral inactivation. The pre-incubation of the viral particles with ethanol or DMSO resulted in a slight decrease in viral infectivity for most of the concentrations tested except for the C18:0 control which decreased 5 log at 6 h of incubation. But as C18:0 inactivated ZIKV in less than 4 h of incubation (when the solvent did not), these results suggest an antiviral effect of C18:0.

## 4. Discussion

Since the 2007 outbreak, it has repeatedly been suggested that ZIKV can be transmitted to newborns through breastfeeding [12]. However, since several case reports described no transmission of ZIKV from infected mothers to their newborn [26], biological factors regulating the efficiency of this route of transmission might exist (viral load, viral form, immunological status, stage of lactation, breast milk composition, etc.). We investigated the role of breast milk and its components in viral transmission using a well-described animal model for ZIKV infection: A129 mice.

ZIKV has previously been shown, by our team [28] and Regla-Nava et al. [32], to disseminate to the mammary glands of infected mice. Here, we investigated the ability of ZIKV to be excreted in breast milk. High viral titers (10^9^ PFU/mL) were detected in the breast milk of infected breastfeeding dams. Between the 12 milk samples tested, viral titers ranged from 6.33 × 10^4^ to 1.08 × 10^9^ PFU/mL of milk. Infectious particles had also been detected by Regla-Nava et al. in the breast milk of AG129 mice after amplification on Vero cells [32]. To better characterize the milk-associated virus, we separated different milk fractions. The cream and whey fractions were separated from the cell pellet by centrifugation and further isolated by several spin-wash cycles. Infectious viral particles were found in both fractions at 10^7^ and 10^9^ PFU/mL, respectively. These results demonstrate that infectious cell-free viral particles are present in breast milk, thus risking transmission to the offspring. Several mechanisms have been suggested to explain the origin of the viral particles in breast milk [12]: Free viral particles in breast milk could have been excreted by mammary luminal epithelial cells as they produce milk and are permissive to ZIKV infection [28]. Although it is less likely, viral particles could also be locally produced in breast milk by infected cells.

The cell pellet was also tested to investigate the ability of breast milk cells to transmit ZIKV. Infectious plaque forming units were detected when co-culturing milk-cells from infected dams with permissive Vero cells (whereas no plaques were observed using milk-cells from MOCK-infected mothers), suggesting the existence of cell-associated virus within breast milk.

To describe more accurately the cell content of the breast milk and thus identify the potentially infectious cell types, we performed flow cytometry experiments using epithelial (CD24) and immune (CD45) cell markers. We observed a majority of epithelial cells ranging from 74.4% to 99% versus an average of 1.28% of CD45-positive cells in breast milk from infected dams, comparable to values observed in breast milk from MOCK dams. These results, obtained using murine milk, are comparable to studies performed on human milk [16]: Witkowska-Zimny et al. detected from 60% to 98% of epithelial cells in mature human milk and less than 2% of immune cells. As the breast milk composition fluctuates during lactation, the epithelial–immune cell ratio varies over time. As described previously, the colostrum is rich in immunological factors whereas the transitional and mature milks are rich in nutritious elements. Here, in mature milk, we further characterized the epithelial cells to determine whether luminal, myoepithelial or progenitor cells from the mammary gland are found in breast milk. Using the CD49f marker, we showed that murine breast milk of ZIKV-infected mice contains mature luminal cells (74.8%) and immature progenitors (4.28%), but no clear population of myoepithelial cells was detected. These results seem coherent with the structure of the mammary epithelium as luminal and progenitor cells are in contact with the lumen of the lactiferous ducts while myoepithelial cells are found beneath the tight luminal cell monolayer [33]. As luminal mammary cells are permissive to ZIKV [28] and predominant in murine breast milk, they could be infected or carry adsorbed ZIKV particles at their surface in the milk. According to a mechanism we suggested [12], infected mammary luminal cells could detach from the mammary epithelium, thus introducing cell-associated ZIKV into the breast milk.

The transmission of ZIKV to breastfed pups was evaluated in the spleens and revealed a very high rate of viral transmission (>90%), with splenic viral loads ranging from 0.93 to 4 × 10^2^ vRNA copies/mg. The viral loads remain relatively low, most probably due to the very early stage of the pups’ infection [13].

According to our previous studies, ZIKV is produced in mouse mammary glands with a peak at 6 days post-subcutaneous infection [28]. Because we observed a reduction in milk production as early as day 5, breast milk samples had to be collected at day 4. As the dams were euthanized after harvesting as much breast milk as possible, pup spleens had to be collected at day 4 as well. However, to optimize the study of the transmission during breastfeeding and detect higher viral loads, pup spleens should be harvested a few days after the peak of production and excretion of ZIKV by the mammary gland. Previously, we already demonstrated that ZIKV is efficiently transmitted during breastfeeding to 1- to 3-day-old pups [13]. Here, we show that older pups are infected through breastfeeding as well (up to 11 days old), even as the impermeability of the intestinal barrier increases [34].

To understand which biological factors could regulate the transmission of ZIKV during breastfeeding, we investigated the effect of several antiviral factors of breast milk, such as LF, LA, and free fatty acids, on ZIKV infectivity. No antiviral effect of LF or LA was observed on ZIKV, although they have been shown to decrease viral infectivity of multiple viruses, such as HIV-1 and CMV [12,35]: LF prevents viral adsorption at the cell membrane, either by interacting with the viral particle (e.g., with gp120 of HIV-1 [36]) or with the target cells (e.g., with heparins [37]). The effect of LF and LA on ZIKV target cells was also investigated (Supplementary Figure S4) but no antiviral effect was observed at any of the physiological concentrations tested. Free fatty acids were shown to inactivate enveloped viruses by interfering with the viral envelope [38]. Here, we studied their effect on ZIKV to better understand the antiviral mechanism of breast milk stored at 4 °C. Both the saturated fatty acid C18:0 and unsaturated fatty acids C18:1, C18:2, and C18:3 exerted an antiviral effect against ZIKV at physiological concentrations. These results confirm the hypothetical mechanism of viral inactivation in refrigerated or frozen breast milk suggested by Pfaender et al. [24]. Indeed, they suggested that upon refrigeration, the MFGM becomes altered, thus exposing the triglyceride core to lipases present in breast milk. The free fatty acids produced would then alter the viral envelope integrity. However, as breast milk storage is responsible for the increase in free fatty acids [24], fresh breast milk does not have an antiviral effect, as demonstrated by Conzelmann et al. [25]. Finally, free fatty acids did not decrease the infection when target cells were treated before viral adsorption, thus showing no effect on the cell receptors or on the early steps of the viral cycle.

## 5. Conclusions

ZIKV transmission through breastfeeding has been described in several cases in past years but is not systematic. In previous studies, we described the ability of ZIKV to cross the intestinal barrier of breastfed pups and to be transmitted by breastfeeding to very young pups (1 to 3 days old) [13]. Here, we show that even older pups (up to 11 days old) are also very susceptible to infection through breastfeeding. In order to understand the biological factors regulating this transmission, we performed in vivo infections of breastfeeding A129 dams and showed that ZIKV is excreted into breast milk as both cell-free and cell-associated virus. Among the different biological factors we investigated (LF, LA, and free fatty acids), the free fatty acids C18:0, C18:1, C18:2, and C18:3 were shown to exert an antiviral effect. As the free fatty acid content in breast milk varies with nutrition, these results should be considered in risk assessment of ZIKV transmission through breastfeeding in order to better advise infected mothers.

## Figures and Tables

**Table 1:** Free fatty acids were purchased from Sigma and resuspended at the following concentrations either in ethanol or DMSO.

Fatty Acid	Stock Concentration (mg/mL)	Solvent	Reference
C10:0	30	Ethanol	Sigma C1875
C14:0	12	DMSO	Sigma M3128
C18:0	22	Ethanol	Sigma S4751
C18:1	100	DMSO	Sigma O1008
C18:2	100	DMSO	Sigma L1376
C18:3	100	DMSO	Sigma L2376

**Table 2:** ZIKV (10^6^ PFU) was incubated either with DMEM-2% FBS, 4% PFA, or with the following non-cytotoxic concentrations of free fatty acids.

Fatty Acid	Assay Concentration (mg/mL)	Condition
C10:0	0.6	Physiological [29]
C14:0	2	Physiological [29]
C18:0	2	Physiological [29]
C18:1	4	Maximum non-cytotoxic
C18:2	3.5	Maximum non-cytotoxic
C18:3	0.5	Physiological [29]

## Supplementary Materials

The following supporting information can be downloaded at: https://www.mdpi.com/article/10.3390/v14050851/s1, Figure S1: Effect of free fatty acids on the cell viability.; Figure S2: Effect of Lactoferrin and lactalbumin on the cell viability.; Figure S3: Control of the effect of the solvents on ZIKV.; Figure S4: Effect of Caco-2 incubation with fatty acids, lactoferrin or lactalbumin before infection.
