# Supplementary material for: Excretion of Cell-Free and Cell-Associated Zika Virus into Breast Milk of Infected Dams and Identification of Antiviral Factors
Source: Viruses. 2022 Apr 20;14(5):851. doi: 10.3390/v14050851 (PMC9144681; doi:10.3390/v14050851)
Supplement: Supplementary file 1 [file viruses-14-00851-s001.zip › FigureS1.pdf]

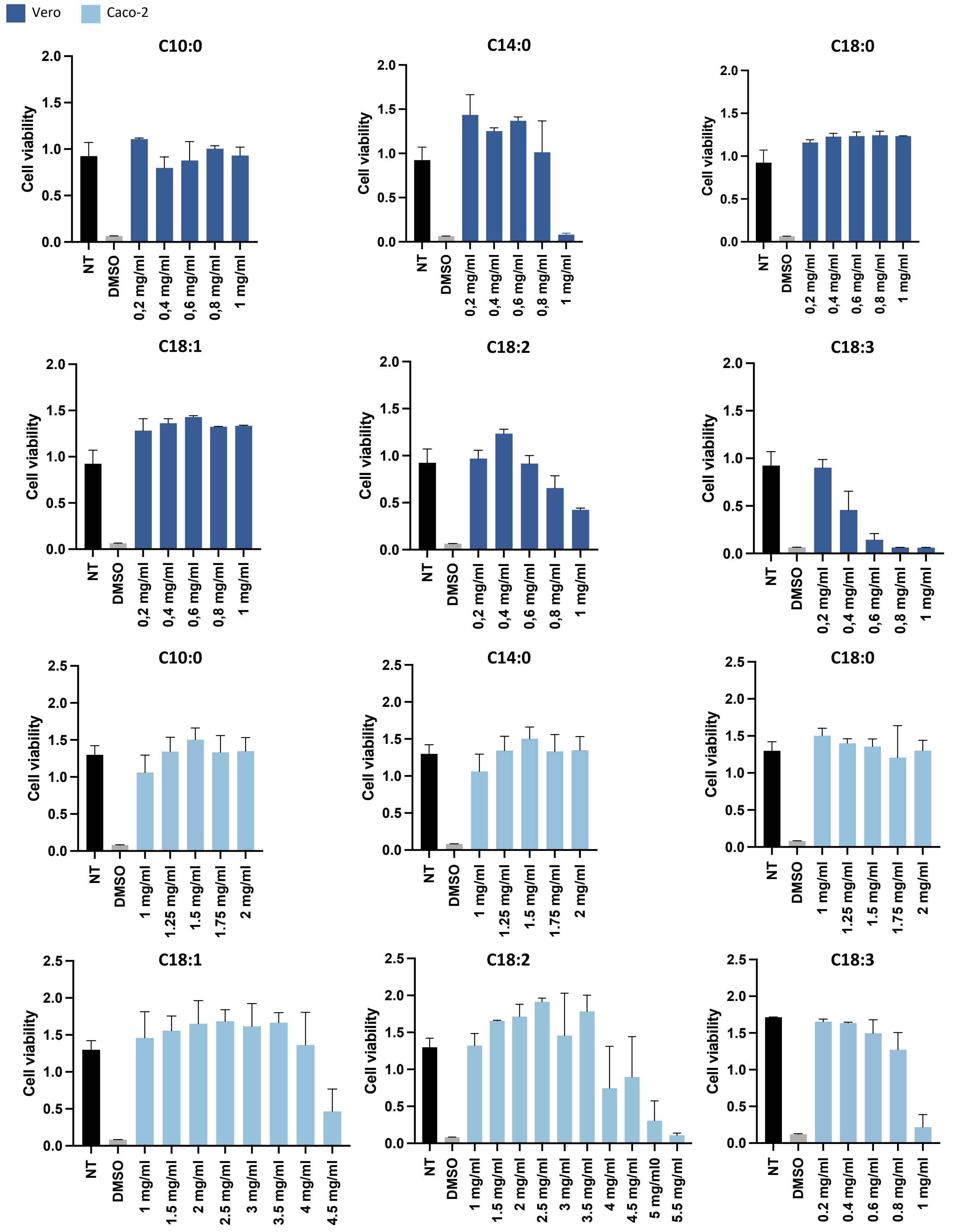

Supplementary figure S1 : Effect of free fatty acids on the cell viability.

Vero (dark blue) or Caco-2 (light blue) cells were incubated with culture medium (NT), dimethyl sulfoxyde (DMSO) or different concentrations of free fatty acids for 2h at 37°C. Cell layers were washed in culture medium and cultured 48h at 37°C before the MTT assay (TC7 : n=3, Vero : n=2).
