# Supplementary material for: Excretion of Cell-Free and Cell-Associated Zika Virus into Breast Milk of Infected Dams and Identification of Antiviral Factors
Source: Viruses. 2022 Apr 20;14(5):851. doi: 10.3390/v14050851 (PMC9144681; doi:10.3390/v14050851)
Supplement: Supplementary file 1 [file viruses-14-00851-s001.zip › FigureS3_S4.pdf]

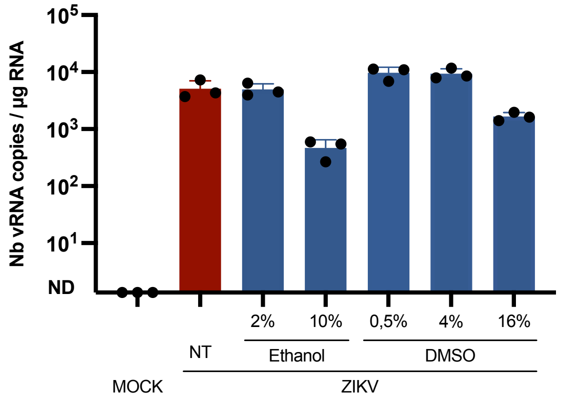

Supplementary figure S3 : Control of the effect of the solvents on ZIKV.

ZIKV particles of the Brazil/2016 strain were incubated with culture medium (NT) or different concentrations of ethanol or dimethyl sulfoxide (DMSO) for 1h at 37°C before infection of Caco-2 cells (MOI 1). At 48h post-infection, cell layers were washed twice in PBS. After intracellular RNA extraction, viral RNAs were amplified by RT-qPCR (n=3).

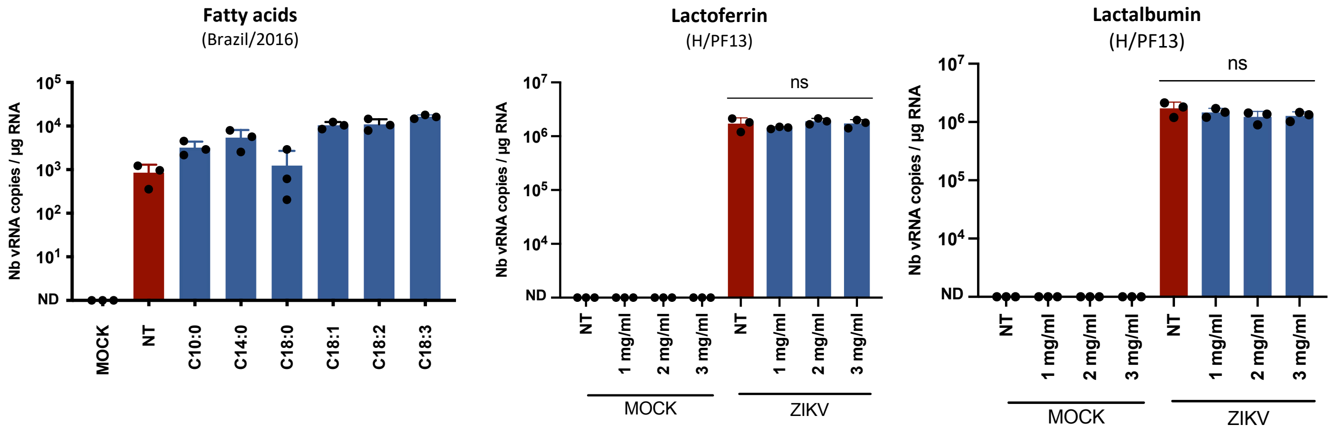

Supplementary figure S4 : Effect of Caco-2 incubation with fatty acids, lactoferrin or lactalbumin before infection.

Caco-2 cells were incubated with culture medium (NT) or different physiological concentrations of free fatty acids, lactoferrin or lactalbumin for 1h at 37°C before ZIKV infection (MOI 1). At 48h post-infection, cell layers were washed twice in PBS. After intracellular RNA extraction, viral RNAs were amplified by RT-qPCR (n=3). ns: Non significantly different by Kruskal-Wallis test ($P<0.05$).
